# Association Between Metabolic Dysfunction-Associated Steatotic Liver Disease and Clinical and Metabolic Outcomes in Patients With Inflammatory Bowel Disease: A Systematic Review and Meta-Analysis

**DOI:** 10.7759/cureus.100043

**Published:** 2025-12-24

**Authors:** Anas Hatab, Noor Gaafar Mansour, Ahmed Rabha, Rawan Abouhatab

**Affiliations:** 1 Gastroenterology, Royal Blackburn Teaching Hospital, Manchester, GBR; 2 General Practice, Northwick Park Hospital, London, GBR; 3 Internal Medicine, Royal Blackburn Hospital, Lancashire, GBR; 4 Gastroenterology, Lancashire Teaching Hospital, Preston, GBR

**Keywords:** diabetes mellitus, hypertension, inflammatory bowel disease, ldl-cholesterol, masld, meta-analysis

## Abstract

Metabolic dysfunction-related steatotic liver disease (MASLD) frequently coexists with inflammatory bowel disease (IBD), yet its metabolic and clinical implications remain unclear. This systematic review and meta-analysis investigated the association between MASLD and metabolic as well as IBD-related outcomes in patients with IBD. A comprehensive search of PubMed, Scopus, Google Scholar, and the Cochrane Library identified observational studies comparing IBD patients with and without MASLD. Four studies comprising 1,109 patients were included. Pooled analyses using fixed- and random-effects models showed that MASLD was significantly associated with higher odds of hypertension (odds ratio (OR) = 2.60; 95% CI: 1.76-3.85; p < 0.00001; I² = 18%) and diabetes mellitus (OR = 12.18; 95% CI: 3.37-44.10; p = 0.0001; I² = 70%), but not with IBD-related surgery (OR = 1.34; 95% CI: 0.89-2.02; p = 0.16; I² = 0%). No significant differences were found in total cholesterol (p = 0.10) or low-density lipid (LDL)-cholesterol (p = 0.07) between groups. Heterogeneity was low to moderate. In conclusion, MASLD in patients with IBD is associated with an increased risk of hypertension and diabetes but not with altered lipid profiles or need for IBD-related surgery, suggesting that MASLD is primarily associated with metabolic comorbidity rather than modification of bowel disease severity.

## Introduction and background

Inflammatory Bowel Disease (IBD) is a disease that has emerged as a major source of morbidity around the world. Primarily manifesting as Crohn’s Disease and Ulcerative Colitis, it causes chronic inflammation of the gastrointestinal tract and a plethora of sequelae downstream of this [1]. Beyond the tissue destruction that is caused by the IBD phenotype, a considerable psychological and financial toll is incurred by the patient as well. The aetiology of IBD is multifactorial, arising from immune dysregulation, genetic predisposition, as well as intestinal microbiome alterations and environmental effects [2].

IBD affects approximately 6.8 million people worldwide and is a major source of global disease burden, with an increasing incidence in both Western and newly developed countries [2]. Although historically common in high-income Western countries, the disease has become more prevalent in Asia, the Middle East, and Latin America, likely because of urbanization, dietary changes, and environmental influences [3]. The relapsing-remitting nature of IBD negatively impacts a patient’s health-related quality of life (HRQoL)-across physical, mental, and social domains, with disease flares and hospitalizations associated with higher rates of anxiety, depression, and reduced work productivity [4]. In addition, direct medical costs, surgery, and loss of productivity have been demonstrated to be substantial [5].

IBD extends beyond the intestines as a systemic disorder that frequently presents with extraintestinal manifestations (EIMs). These can affect nearly any organ system, with a lifetime prevalence ranging from 5% to 50% of patients [6]. Commonly involved systems include the musculoskeletal (arthritis), dermatological (erythema nodosum, pyoderma gangrenosum), ocular (uveitis, episcleritis), and hepatobiliary (primary sclerosing cholangitis, steatotic liver disease) [7]. These EIMs can often overlap with intestinal inflammation, or they can be present on their own, further complicating the disease. The appearance of EIMs is tied to greater morbidity, decreased quality of life, and increased healthcare utilization, thus IBD care must be multidisciplinary [8].

Metabolic dysfunction-associated steatotic liver disease (MASLD), formerly known as NAFLD, is the most prevalent chronic liver condition worldwide, affecting nearly 30% of the world's population [9]. The nomenclature change underscores its pathogenesis with conditions rooted in metabolic dysregulation; namely, insulin resistance, obesity, type 2 diabetes, and atherogenic dyslipidemia. Simple steatosis and the more severe metabolic dysfunction-associated steatohepatitis (MASH), which can develop into fibrosis, cirrhosis, and hepatocellular carcinoma (HCC), are just two examples of the disease's progressive spectrum of liver damage [10]. Beyond hepatic effects, MASLD is a systemic disorder linked to cardiometabolic events, with approximately 75% of adults with type 2 diabetes exhibiting comorbid MASLD, thus highlighting their bidirectional relationship [11].

It is thought that metabolic dysregulation, pro-inflammatory mediators, and genetic polymorphisms interact intricately to cause MASLD, which in turn causes hepatic steatosis. Insulin resistance, a central feature of MASLD pathogenesis, promotes lipogenesis and impairs β-oxidation, contributing to triglyceride accumulation, reactive oxygen species generation, and oxidative stress-induced hepatocyte mitochondrial injury [12]. This is further compounded by changes in gut-derived signalling molecules to the liver, increased secretion of pro-inflammatory cytokines, and genetic variants at key loci such as PNPLA3 and TM6SF2 that affect lipid metabolism and extracellular matrix degradation [13]. Of all histologic characteristics, the extent of fibrosis is the most important prognostic factor for both future decompensation and survival [14]. With few therapeutic options available (resmetirom being the only drug recently approved by the FDA for MASH), management of MASLD remains centered around lifestyle modifications to induce weight loss and management of associated comorbidities [15].

The interconnection between IBD and MASLD involves three overlapping pathophysiological mechanisms: chronic low-grade inflammation, immune system dysregulation, and gut-liver axis dysregulation. Both conditions share dysbiosis, barrier dysfunction, and metabolic disturbance. The dysbiosis of IBD leads to increased mucosal permeability, allowing bacterial products such as endotoxins (lipopolysaccharide (LPS)) to be delivered to the portal system to promote hepatic steatosis and necro-inflammation [16]. Chronic stimulation of toll-like receptors and chronic activation of Kupffer cells promote lipogenesis and fibrosis, which consequently creates a positive inflammatory feedback loop between the gut and liver. Furthermore, the systemic inflammatory environment present in IBD with elevated cytokines like tumor necrosis factor (TNF)-α, interleukin (IL)-6, and IL-1β further contributes to peripheral insulin resistance and increased hepatocyte metabolic stress, predisposing to an increased risk of MASLD [17].

While the pathophysiology of inflammatory bowel disease (IBD) is fundamentally rooted in a dysregulated immune response to intestinal microbiota within a genetically susceptible host, its involvement in metabolic-associated steatotic liver disease (MASLD) introduces a secondary layer of metabolic and hepatic stress amplified by gut-derived signals [18]. In standalone IBD, the core mechanisms involve genetic factors (e.g., mutations in *NOD2* or *ATG16L1* genes that impair bacterial sensing and autophagy) [19], environmental triggers (such as diet, smoking, or infections), and epithelial barrier defects that allow microbial antigens to penetrate the mucosa. This triggers an aberrant innate and adaptive immune cascade, characterized by overactivation of T-helper cells (Th1/Th17 in Crohn’s disease, Th2 in ulcerative colitis), recruitment of inflammatory monocytes, and sustained production of pro-inflammatory cytokines like TNF-α, IL-6, IL-1β, and IL-23 [20]. The result is chronic gut inflammation, often manifesting as transmural lesions in Crohn’s disease or superficial mucosal ulceration in ulcerative colitis, with symptoms driven by localized tissue damage and impaired wound healing. 

In contrast, when IBD pathophysiology intersects with MASLD, the gut-centric immune dysregulation extends systemically via the gut-liver axis, transforming IBD from a primarily intestinal disorder into a contributor to hepatic metabolic pathology [2]. MASLD itself is predominantly metabolic in origin, arising from insulin resistance, dyslipidemia, and adipose tissue overflow that promote intrahepatic triglyceride accumulation (steatosis), oxidative stress, mitochondrial dysfunction, and Kupffer cell activation leading to necroinflammation and fibrosis [21]. Unlike IBD’s immune primacy, MASLD emphasizes multifactorial drivers like genetic polymorphisms (e.g., PNPLA3 variants), endocrine imbalances, and gut microbiota alterations that disrupt lipid homeostasis without initial overt inflammation [22]. However, in IBD patients, this metabolic vulnerability is exacerbated by IBD-specific factors: increased intestinal permeability allows the translocation of bacterial products (e.g., LPS) into the portal circulation, directly stimulating hepatic toll-like receptors (TLRs) and fostering a pro-fibrotic environment distinct from pure metabolic MASLD [16] . Additionally, IBD’s systemic cytokine storm promotes peripheral insulin resistance and visceral adiposity-even in non-obese individuals-accelerating hepatocyte lipid deposition and progression to steatohepatitis (MASH), which is less common in isolated MASLD without inflammatory comorbidities [9]. 

There are key differences that lie in disease localization and initiation. IBD originates in the gut with immune-mediated triggers, whereas MASLD is liver-focused and metabolically initiated, but their overlap creates a bidirectional vicious cycle where IBD-induced dysbiosis reduces protective short-chain fatty acids (SCFAs) and indoles, further impairing hepatic barrier function and amplifying metabolic stress [23]. This interaction is particularly pronounced in Crohn’s disease compared to ulcerative colitis, with IBD patients showing higher MASLD prevalence independent of traditional metabolic risks, leading to worsened outcomes like increased fibrosis risk and cardiovascular complications [24]. Understanding these contrasts highlights the need for integrated management targeting both gut inflammation and hepatic metabolism in comorbid cases.

Microbial and metabolic drivers have been identified as a recent development contributing to the co-pathogenesis of IBD and MASLD. The identification of liver steatosis in non-obese IBD patients led to the recognition of subclinical inflammation, dyslipidemia, and an increase in visceral adiposity (but not total adiposity) as the main drivers [18]. A reduction in health-promoting microbial signals (e.g., indole derivatives and short-chain fatty acids) is present in both diseases [25]. Consequently, gut microbial imbalance promotes hepatic steatosis, inflammation, and fibrogenesis through metabolic and immune pathways. The coexistence of MASLD in IBD patients has been further associated with increased cardiovascular risk, greater disease activity, and poorer overall clinical outcomes [26].

Review objective 

This study aims to evaluate and quantify the clinical impact of metabolic dysfunction-associated steatotic liver disease (MASLD) in patients with inflammatory bowel disease (IBD). Specifically, we seek to assess MASLD’s effects on (1) metabolic comorbidity burden, (2) selected biochemical pathways, and (3) disease course, while correlating these with laboratory markers and IBD activity indices.

Methods 

Search Strategy

A literature search was conducted from database inception through November 14, 2025, across PubMed, Scopus, Web of Science, Embase, and the Cochrane Library for studies reporting MASLD prevalence in adult IBD patients. MeSH terms were combined with entry terms including “metabolic dysfunction-associated steatotic liver disease,” “MASLD,” “hepatic steatosis,” “nonalcoholic fatty liver disease,” “Crohn’s disease,” “ulcerative colitis,” “inflammatory bowel disease,” “metabolic syndrome,” “dyslipidemia,” “insulin resistance,” and “liver fibrosis.” The list of references in eligible studies and relevant meta-analyses was manually reviewed for additional publications.

Inclusion Criteria

Studies were restricted to observational designs (cross-sectional or cohort) comparing MASLD and non-MASLD groups in adult IBD populations. MASLD diagnosis required radiologic evidence of hepatic steatosis plus at least one metabolic risk abnormality consistent with current definitions. Only studies providing extractable quantitative data on clinical, metabolic, or hepatic outcomes, available in full-text English, were included.

Exclusion Criteria

Exclusions applied to studies lacking direct MASLD vs. non-MASLD comparisons, unreportable outcome data, unvalidated MASLD criteria, pediatric or mixed populations without adult IBD stratification, or non-primary research (e.g., abstracts, reviews, commentaries). Single-arm studies and those with overlapping cohorts were also excluded.

Outcome Measures

Primary outcomes encompassed these clinical endpoints: hypertension prevalence, diabetes mellitus prevalence, and IBD-related surgery. IBD-related surgery was defined as any history (lifetime prevalence) of major intestinal resection or IBD-specific surgical procedure (colectomy, ileocecal resection, stricturoplasty, fistula/abscess surgery, etc.). The analysis reflects cumulative surgical burden up to the time of MASLD assessment rather than incident surgeries occurring after MASLD diagnosis. Consequently, no temporal or causal relationship between MASLD and the need for future surgery can be established from the current data. Secondary outcomes included metabolic and hepatic parameters such as serum lipids (total cholesterol, LDL, high-density lipid (HDL), triglycerides), fasting glucose and insulin, insulin resistance indices (homeostatic model assessment of insulin resistance (HOMA-IR), triglyceride-glucose index (TyG)), and liver markers (alanine aminotransferase (ALT), aspartate aminotransferase (AST), gamma-glutamyl transferase (GGT), fibrosis index based on 4 factors (FIB-4), controlled attenuation parameter (CAP), liver stiffness. IBD characteristics, including duration, subtype, and activity, were extracted when available to contextualize metabolic and hepatic findings.

Data Extraction and Quality Assessment

A standardized form was used to collect study-level data on design, population characteristics, MASLD ascertainment, metabolic variables, hepatic fibrosis assessment, and IBD-specific outcomes. Data were independently extracted by two reviewers, with discrepancies resolved through consensus. Quality was assessed using the Newcastle-Ottawa Scale (NOS), evaluating selection, comparability, and outcome domains, with risk of bias classified as low, moderate, or high based on predefined cutoffs [27].

Statistical analysis

Continuous outcomes were analyzed as standardized mean differences (SMDs), while dichotomous outcomes were pooled as odds ratios (ORs) with 95% CIs. Fixed-effects models were used for low heterogeneity (I² < 50%); random-effects models were applied otherwise. Publication bias was not assessed formally due to the limited number of included studies. Analyses were performed using Review Manager 5.4 (Cochrane, London, UK) [28], with p < 0.05 considered statistically significant.

## Review

Results 

Search Result and Study Selection

**Figure 1:**
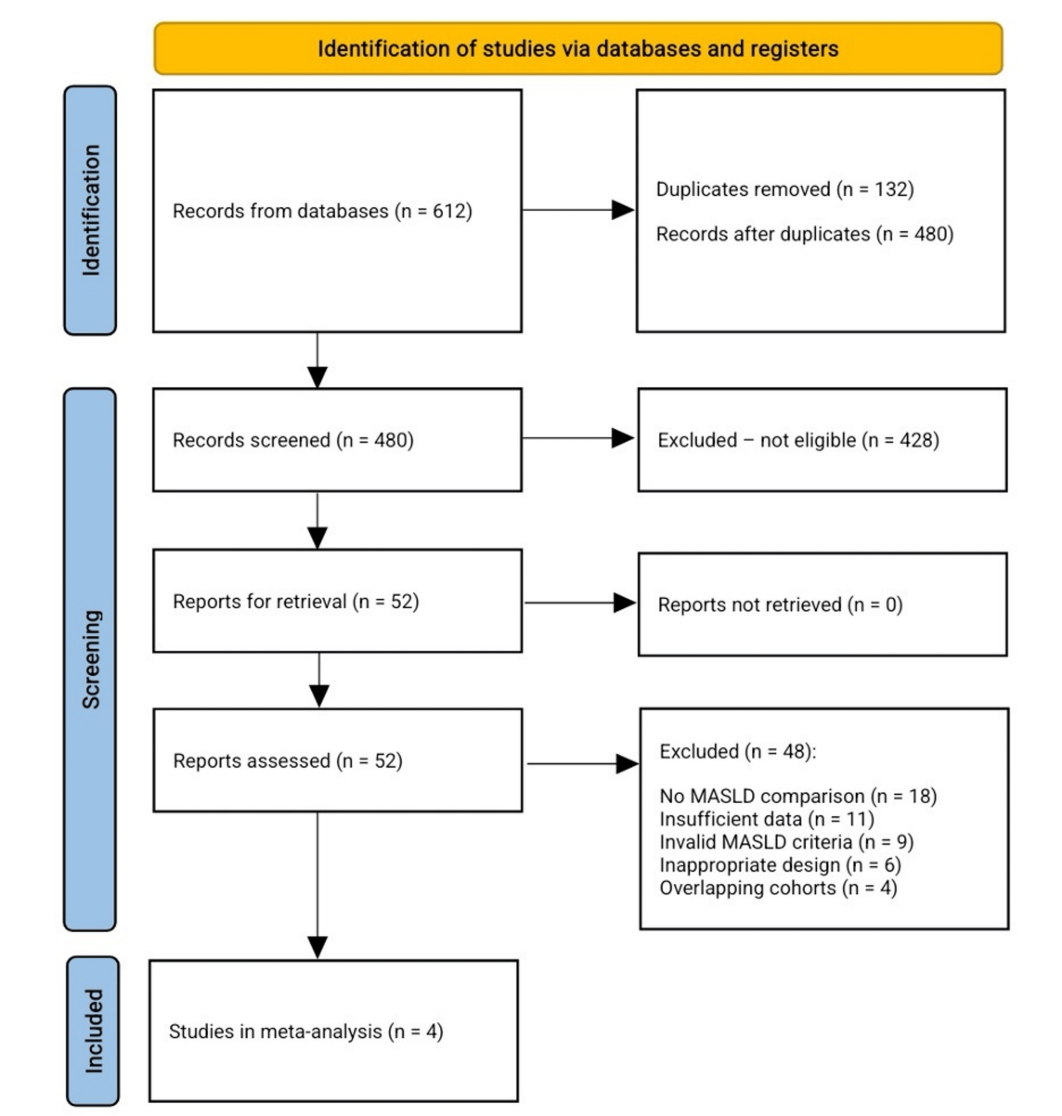
PRISMA flow chart for the included studies PRISMA: Preferred Reporting Items for Systematic Reviews and Meta-Analyses

The initial database search yielded 612 unique citations. After removing 132 duplicates, 480 underwent title and abstract screening, of which 428 were excluded for failing eligibility criteria (e.g., non-comparative designs, lack of MASLD analysis, inappropriate populations, or absent outcomes). Fifty-two full-text manuscripts were reviewed, and 48 were excluded due to unvalidated MASLD diagnoses, insufficient reporting, lack of IBD stratification, methodological flaws, or overlapping data. Four studies met all criteria and were included in the quantitative synthesis, providing comparative data on metabolic, hepatic, and IBD-specific outcomes. The screening process is summarized in Figure 1.

Study Characteristics

The meta-analysis evaluated the effects of metabolic dysfunction-associated steatotic liver disease (MASLD) versus non-MASLD in adult IBD patients, drawing from four observational studies with retrospective cohort or cross-sectional designs in tertiary care settings. Despite geographic diversity (Italy, Spain, Taiwan, Brazil), populations exhibited similar IBD subtype distributions and demographics. The pooled cohort comprised 704 patients (214 with MASLD, 490 without), including 373 males and 331 females.

MASLD was defined consistently, requiring radiologic hepatic steatosis and ≥1 cardiometabolic risk factor. Imaging modalities varied: two studies used abdominal ultrasound, one combined ultrasound with controlled attenuation parameter (CAP), and one relied primarily on CAP via FibroScan. Metabolic criteria uniformly included elevated BMI or waist circumference, dyslipidemia, insulin resistance, impaired fasting glucose, or hypertension.

MASLD patients were consistently older, with higher BMI, central adiposity, and metabolic syndrome clustering compared to non-MASLD counterparts. Studies reported elevated triglycerides, fasting glucose, insulin, γ-glutamyltransferase, and insulin resistance indices in MASLD groups. Fibrosis assessment varied, incorporating elastography-based liver stiffness and serum markers (e.g., FIB-4), with MASLD linked to increased advanced fibrosis risk. No study associated MASLD with IBD-related surgery, emphasizing metabolic over luminal drivers. 

This heightened cardiometabolic burden is consistently supported across studies. Abenavoli et al. [29] reported elevated blood pressure, triglycerides, and BMI in MASLD cohorts, while García-Mateo et al. [30] identified increased carotid intima-media thickness and subclinical atherosclerosis. The present meta-analysis corroborates these observations with a pooled odds ratio for hypertension of 2.60 (95% CI 1.76-3.85), aligning closely with Stafie et al. [31], who identified hypertension as an independent predictor of MASLD (OR 2.77). Indeed, in the 2024 study by Oliveira et al. [32], the prevalence of metabolic dysfunction-associated steatotic liver disease (MASLD) within the IBD population investigated was 44.3% (Table 1).

**Table 1:** Summary of study characteristics and parameters The table summarizes study characteristics, including sample size, patient demographics, metabolic dysfunction-related steatotic liver disease (MASLD) diagnostic methods, and primary clinical and metabolic outcomes in studies comparing MASLD and non-MASLD in patients with inflammatory bowel disease (IBD). BMI: body mass index; WC: waist circumference; LAP: lipid accumulation product; VAI: visceral adiposity index; CAP: controlled attenuation parameter; LSM: liver stiffness measurement; CIMT: carotid intima-media thickness; CD: Crohn’s disease; UC: ulcerative colitis.

Variable	Abenavoli et al. [29]	García-Mateo et al. [30]	Hsiao et al. [33]	Oliveira et al. [32]
Study Design & Setting	Retrospective observational; single-center Italy; 2021–2024	Cross-sectional matched cohort; Spain; 2020–2021	Retrospective cross-sectional; Taiwan; 2019–2023; IBD in remission only	Cross-sectional; Brazil; 2019–2021
Sample Size (MASLD / Non-MASLD)	67/277 (+ NAFLD subgroup n=14)	50/50	35/85	62/78 (n=140)
Patient Demographics	Age 52±12 vs 46±16; Male 72% vs 54%; BMI 27±4 vs 24±4; WC 100±11 vs 90±11	Mean age ~50 y; 52% female; MASLD had higher BMI & inactivity	Median age 43.5; 67.5% male; BMI 26.3 vs 21.5; WC 93 vs 76	Age 53.5±11.9 vs 46.6±14.3; 67% female; CD 63.6%; disease 9.7±7.9 yrs
MASLD Definition & Diagnostic Tools	US-detected steatosis + ≥1 metabolic risk criterion	Ultrasound + CAP >248 dB/m + metabolic factor	CAP ≥248 dB/m + Asian metabolic criteria	Imaging (US/CT/MRI) steatosis + ≥1 metabolic risk factor
IBD Characteristics	CD 33% vs 35%; UC 67% vs 65%; duration 14±11 vs 12±11 yrs; no activity difference	CD 51%, UC 49%; ileocolonic 43%; UC extensive 59%; low activity	CD 45, UC 75; shorter IBD duration in MASLD (2.8 vs 5.3 yrs)	CD is more common; active disease 60%; longer duration in MASLD (11.6 vs 8.3 yrs)
Metabolic & Biochemical Findings	↑ALT (27 vs 19), ↑AST (23 vs 20), ↑TG (118 vs 95), ↓HDL (49 vs 57), ↑glucose, ↑insulin; ↑HOMA-IR, METS-IR, TyG	↑CRP; atherogenic lipoproteins; abnormal dense LDL subclasses	↑ALT (24 vs 17), ↑GGT (24 vs 13), ↑TG, ↓HDL, ↑HOMA-IR, ↑TyG	↑BMI, ↑waist, ↑MS components (2.9 vs 1.6); worse metabolic profile
Fibrosis Assessment	FIB-4 (no significant difference); AUC 0.562	No TE fibrosis staging; evaluated CIMT + carotid plaques	Significant fibrosis (LSM ≥7.2 kPa): 17.1% vs 2.4%; p=0.008	FIB-4: advanced fibrosis 6.5%; excluded in 63.5%
Cardiometabolic Risk Findings	↑T2DM (13% vs 3%), ↑HTN (36% vs 13%), ↑obesity indices (BMI, waist, LAP, VAI)	MASLD is strongly associated with CIMT; ↑metabolic syndrome prevalence	Fibrosis strongly correlated with MASLD; marked metabolic dysfunction	MS components OR 2.20; obesity OR 2.29; waist nearly significant
IBD-Related Surgery	22% vs 16%, p=0.492 (ns)	24.4% vs 19.1%, p=0.551 (ns)	28.6% vs 15.3%, p=0.093 (ns)	No association (21% overall; p=0.730)
Key Statistical Results	Strong metabolic differences; METS-IR AUC 0.754; waist AUC 0.754; LAP 0.737; BMI 0.709; TG/HDL 0.701	MASLD OR 5.05 for altered CIMT; dense LDL predictive; female sex OR 3.32	Predictors: ↑Hb (aOR 1.91), ↑GGT (aOR 1.11), fibrosis (aOR 31.25)	Multivariable AUC 0.85; metabolic drivers dominate; no IBD phenotype effects
Level of Evidence	Level III	Level III	Level III	Level III
Conclusion	MASLD is strongly linked to insulin resistance, dyslipidemia; metabolic indices are predictive	MASLD independently predicts subclinical atherosclerosis; lipoprotein subclass is valuable	MASLD is common in remission; higher fibrosis risk; TE/CAP is essential for screening	MASLD is highly prevalent,; driven by metabolic factors rather than IBD characteristics

Quality Assessment of the Included Studies

Methodological quality was appraised with the Newcastle-Ottawa Scale (NOS), allocating stars within Selection, Comparability, and Outcome domains; aggregate star counts translated into low, moderate, or high quality classifications. Table 2 itemizes the individual NOS ratings for each retained investigation.

**Table 2:** Quality assessment of included studies using the Newcastle–Ottawa Scale (NOS) * = low score for the respective domain; ** = moderate score, acceptable quality; *** = high score, strong quality in the respective domain.

Study	Selection	Comparability	Outcome	Total score (out of 9)
Abenavoli et al. [29]	**	*	**	6
García-Mateo et al. [30]	***	***	**	7
Hsiao et al. [34]	***	***	***	8
Oliveira et al. [32]	***	***	**	7

Results of the meta-analysis 

Hypertension Prevalence in IBD Patients With vs. Without MASLD

**Figure 2:**
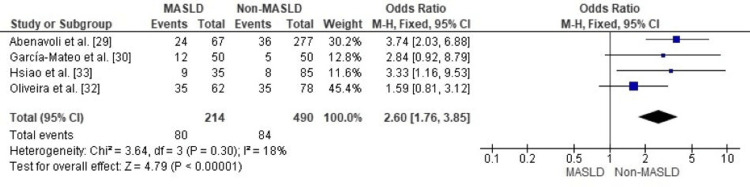
: Forest plot comparing MASLD and non-MASLD groups for prevalence of hypertension. MASLD: metabolic dysfunction–associated steatotic liver disease; OR: odds ratio; CI: confidence interval. Source: [29,30,32,33].

In patients with inflammatory bowel disease (IBD), the prevalence of hypertension was significantly higher among those with comorbid metabolic dysfunction-associated steatotic liver disease (MASLD) than in those without MASLD. The pooled analysis showed an odds ratio of 2.60 (95% CI: 1.76-3.85; p < 0.00001), meaning patients with IBD and MASLD had more than 2.5 times the odds of hypertension compared with IBD patients without MASLD (Figure 2).

Heterogeneity among the included studies was low (I² = 18%, p = 0.30), indicating consistent results across different study designs, patient populations, and diagnostic criteria. This low variability strengthens confidence in the robustness and reliability of the observed association.

Comparison of IBD-Related Surgery Between MASLD and Non-MASLD in Patients With Inflammatory Bowel Disease

**Figure 3:**
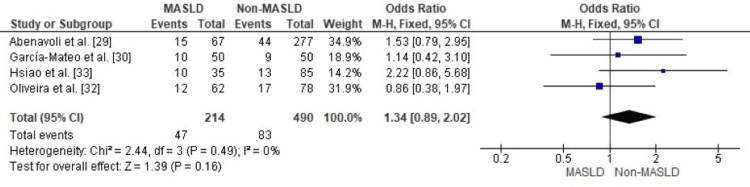
Forest plot comparing MASLD and non-MASLD groups for IBD-related surgery MASLD: metabolic dysfunction–associated steatotic liver disease; OR: odds ratio; CI: confidence interval; IBD: inflammatory bowel disease. Source: [29,30,32].

A meta-analysis of the four studies showed that MASLD did not increase IBD-related surgical risk (OR pooled 1.34, 95% CI 0.89-2.02, p = 0.16) with no significant heterogeneity (I² = 0%, p = 0.49). This result indicates a similarity in the study design and subjects across the datasets and suggests that the lack of an observed increase in surgical risk is unlikely to be due to chance (Figure 3).

Comparison of Diabetes Mellitus Prevalence Between MASLD and Non-MASLD in Patients With Inflammatory Bowel Disease

Diabetes mellitus represents a key cardiometabolic comorbidity of particular interest in patients with inflammatory bowel disease (IBD), given the shared pathways of insulin resistance and systemic inflammation with metabolic dysfunction-associated steatotic liver disease (MASLD).

**Figure 4:**
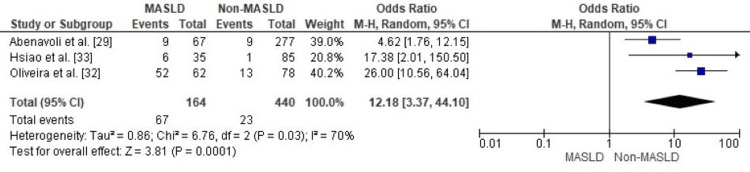
Forest plot comparing MASLD and non-MASLD groups for prevalence of diabetes mellitus MASLD: metabolic dysfunction–associated steatotic liver disease; OR: odds ratio; CI: confidence interval. Source: [29,32].

Forest plot of the meta-analysis showing the prevalence of diabetes mellitus among patients with inflammatory bowel disease, as stratified by the presence of MASLD. A statistically significant positive relationship was identified in the MASLD group, as the random-effects summary estimated an odds ratio of 12.18 (95 % CI 3.37-44.10, p = 0.0001). Substantial heterogeneity was observed (I² = 70%, p = 0.03), reflecting variability among the included studies in terms of population characteristics, metabolic profiles, and diagnostic approaches. Nonetheless, the direction of effect was consistent across studies, supporting a strong association between MASLD and increased diabetes risk among IBD patients (Figure 4).

Comparison of Serum Total Cholesterol Levels Between MASLD and Non-MASLD in Patients With Inflammatory Bowel Disease

**Figure 5:**
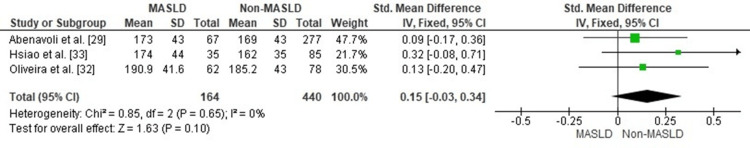
Forest plot comparing MASLD and non-MASLD groups for serum total cholesterol levels MASLD: Metabolic Dysfunction–Associated Steatotic Liver Disease; SMD: Standardized Mean Difference; CI: Confidence Interval. Source: [29,32]

Circulating total cholesterol showed statistical equivalence between IBD cohorts, with a pooled SMD of 0.15 (95% CI: −0.03 to 0.34) under the fixed-effect model. The forest plot (Figure 5) of total cholesterol also revealed no significant difference (p = 0.10) between IBD subjects with or without MASLD. The heterogeneity of effect sizes between the two groups was not significant (I² = 0 %, p = 0.65), suggesting that effect sizes, patient characteristics, and laboratory procedures are similar between different studies. Therefore, there was no difference in total cholesterol level in IBD subjects with and without MASLD (Figure 5).

Comparison of Serum LDL-Cholesterol Levels Between MASLD and Non-MASLD in Patients With Inflammatory Bowel Disease

**Figure 6:**
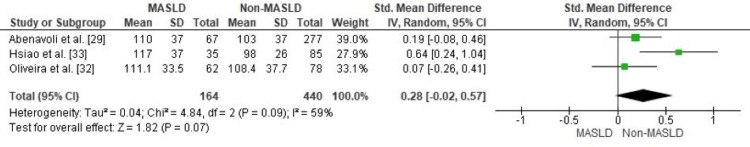
Forest plot comparing MASLD and non-MASLD groups for serum LDL-cholesterol levels MASLD: metabolic dysfunction–associated steatotic liver disease; LDL: low-density lipoprotein; SMD: standardized mean difference; CI: confidence interval. Source: [29,32].

No clinically important difference in LDL-cholesterol levels between IBD patients with and without MASLD was observed on the basis of available data. As illustrated in Figure 6, a random-effects model analysis failed to demonstrate statistical significance and reported a pooled standardized mean difference of 0.28 (95% CI: −0.02 to 0.57). Despite a slightly increased result for the MASLD group, it was non-significant (p = 0.07). A moderate heterogeneity was present (I² = 59%, p = 0.09), which may be a consequence of inter-study differences in lipid phenotype, patient characteristics, and methodology.

Discussion

Our systematic review and meta-analysis establish metabolic dysfunction-associated steatotic liver disease (MASLD) in patients with inflammatory bowel disease (IBD) as a cardiometabolic comorbidity rather than a direct modulator of intestinal disease severity or progression. Pooled analyses demonstrated strong associations with hypertension and diabetes mellitus, but no significant relationship with IBD-related surgical interventions or alterations in total/LDL-cholesterol.

The association with diabetes mellitus was particularly striking (Figure 4), yielding a pooled OR of 12.18 (95% CI 3.37-44.10). Although the confidence interval is wide, reflecting low precision likely attributable to the limited number of included studies (n=4), small sample sizes in some cohorts, and variability in event rates, the direction of the effect remained consistent across all studies, supporting a robust association between MASLD and elevated diabetes risk in IBD patients. This finding is further illustrated in a pilot study by Abenavoli et al. [34], where IBD patients diagnosed with MASLD-requiring hepatic steatosis plus at least one cardiometabolic risk factor had a significantly higher prevalence of type 2 diabetes mellitus (19%) compared to those meeting prior NAFLD criteria without cardiometabolic risks (0%; p=0.045). These observations underscore how the updated MASLD nomenclature more effectively captures patients with metabolic comorbidities, such as hyperglycemia and insulin resistance, thereby identifying those at greater cardiometabolic risk. Elevated HOMA-IR, fasting glucose, and insulin levels in MASLD patients, as reported by Hsiao et al. [34] and Oliveira et al. [32], underscore the role of underlying insulin resistance in driving type 2 diabetes pathogenesis within this population. The substantial heterogeneity observed (I² = 70%, p = 0.03) warrants careful consideration of potential contributing factors. Variability in study populations-such as differences in age distributions, sex ratios, ethnic backgrounds (e.g., Taiwanese in Hsiao et al. [34] vs. Brazilian in Oliveira et al. [32]), IBD subtypes (e.g., Crohn's disease vs. ulcerative colitis), disease duration, and concomitant treatments (e.g., corticosteroid or immunomodulator use)-could account for some of the inconsistency. Additionally, methodological differences in diagnosing MASLD, including reliance on non-invasive techniques such as ultrasound or transient elastography in Abenavoli et al. [29] and Hsiao et al. [34], versus other approaches in García-Mateo et al. [30] and Oliveira et al. [32], may have influenced case ascertainment and introduced bias. Critically, discrepancies in the definition and ascertainment of diabetes mellitus across the four included studies likely played a significant role in the heterogeneity. For instance, in Abenavoli et al. [29], type 2 diabetes mellitus was identified through confirmed clinical diagnosis or ongoing antidiabetic therapy, with fasting glucose ≥100 mg/dL considered as part of the cardiometabolic risk criteria for MASLD. In Hsiao et al. [34], diabetes was ascertained via self-reported medical history without detailed biochemical thresholds. In García-Mateo et al. [30], diabetes (type 2 or prediabetes) was assessed as part of cardiovascular risk profiling, likely based on clinical records and metabolic parameters such as fasting glucose or glycated hemoglobin (HbA1c), though specific cutoffs were not explicitly stated. Similarly, in Oliveira et al. [32], diabetes was evaluated in the context of metabolic risk factors, incorporating biochemical assessments like fasting glucose and HOMA-IR to identify insulin resistance and related states. These divergent approaches-ranging from self-report and clinical history to biochemical markers-could lead to varying prevalence estimates and odds ratios, particularly in IBD cohorts where metabolic profiling may not be standardized. Future meta-analyses could mitigate this by conducting subgroup analyses stratified by diagnostic criteria or employing meta-regression to quantify the impact of these methodological variations. Notwithstanding the heterogeneity, the overarching findings emphasize the need for integrated metabolic screening and management strategies in IBD patients with MASLD to mitigate diabetes risk. The strong relationship with diabetes is further reinforced by Latif and Ahsan [35], who documented a 51.9% increased odds of MASLD in patients with established type 2 diabetes.

Dyslipidemia in MASLD followed a distinctly atherogenic pattern, characterized by significantly higher triglycerides and lower HDL-cholesterol [29,34], alongside an unfavorable non-HDL/HDL ratio. In contrast, total and LDL-cholesterol levels showed no pooled differences, consistent with emerging evidence that remnant cholesterol and lipid ratios-rather than conventional LDL-C-are primary drivers of MASLD risk [36,37]. Despite clear metabolic linkages, MASLD did not exacerbate the IBD clinical course. The pooled OR for IBD-related surgery was non-significant (1.34, 95% CI 0.89-2.02), and individual studies [29-32,38] uniformly demonstrated comparable surgical rates, disease duration, and extraintestinal manifestations, indicating that hepatic steatosis does not amplify mucosal inflammation or structural bowel damage.

Nevertheless, liver disease progression was more advanced in the presence of MASLD: Hsiao et al. [34] reported significant fibrosis (≥7.2 kPa) in 17.1% versus 2.4% of non-MASLD patients; Oliveira et al. [32,39] documented higher FIB-4 scores and histological confirmation of significant fibrosis in 68.7% and metabolic dysfunction-associated steatohepatitis in 25% of biopsies. Systemic inflammatory markers (ALI, CAR) were independently associated with MASLD [40], implicating chronic low-grade inflammation in driving hepatic fibrogenesis among IBD patients.

Taken together, the body of evidence portrays comorbid MASLD in IBD as an amplifier of systemic metabolic and cardiovascular risk rather than acting as a primary driver of gut-specific inflammation. The strikingly high odds of diabetes mellitus (pooled OR >12) and hypertension (pooled OR ≈2.6), coupled with an atherogenic dyslipidemic profile (↑triglycerides, ↓HDL, unfavourable lipid ratios), align with the broader pathophysiological framework of IBD as a systemic inflammatory condition that promotes peripheral insulin resistance, visceral adiposity, and endothelial dysfunction-even in non-obese individuals [2, 18, 19, 24]. Chronic translocation of microbial products and pro-inflammatory cytokines (TNF-α, IL-6, IL-1β) through a leaky gut barrier appears to converge with classic metabolic pathways (insulin resistance, lipotoxicity, oxidative stress), thereby accelerating hepatic steatosis and its progression to steatohepatitis and fibrosis [16, 17, 23, 26].

Notably, the absence of an association between MASLD and IBD-related surgery (although notably the temporal relationship between IBD related surgery and the onset of IBD itself was unclear in many of the aforementioned studies where IBD related surgery was defined as a historical characteristic) or more aggressive intestinal phenotypes suggests that the gut-liver axis in this setting is largely unidirectional: IBD-driven systemic inflammation and dysbiosis promotes liver injury, but established hepatic steatosis does not substantially feedback to worsen mucosal disease activity in the short-to-medium term [29-32,38]. This contrasts with other extra-intestinal manifestations (e.g., primary sclerosing cholangitis), where bidirectional crosstalk is more evident. The accelerated hepatic fibrosis observed in IBD-MASLD cohorts, despite relatively preserved bowel disease course, highlights a clinically silent but prognostically important progression toward advanced liver disease, driven by the synergistic effects of low-grade systemic inflammation and metabolic stress [32,34,39,40].

These findings carry several important clinical and research implications. First, MASLD should be viewed as a cardiometabolic risk multiplier in IBD rather than merely a bystander hepatic manifestation; routine screening with non-invasive fibrosis markers (FIB-4, transient elastography) and targeted cardiometabolic evaluation (HOMA-IR, lipid ratios, blood pressure, glucose tolerance) is warranted, especially in patients with long-standing disease, Crohn’s disease phenotype, or corticosteroid exposure. Second, therapeutic strategies that simultaneously address both axes-anti-inflammatory/biologic therapy for IBD and lifestyle/metabolic interventions (weight loss ≥7-10%, GLP-1 receptor agonists, SGLT2 inhibitors, or emerging MASH-specific agents such as resmetirom)-may yield synergistic benefits for liver and cardiovascular outcomes [15,22]. Finally, the marked heterogeneity in diagnostic criteria and population characteristics underscores the urgent need for large, prospective, multi-ethnic cohort studies using harmonised MASLD/MASH definitions and standardised metabolic phenotyping to clarify temporal relationships, identify high-risk subgroups, and evaluate whether early metabolic intervention can prevent fibrosis progression in this vulnerable population.

In conclusion, MASLD in the context of IBD represents a prototype of inflammation-metabolism interplay, conferring substantial cardiometabolic morbidity and accelerated liver fibrosis without directly worsening intestinal disease severity. Integrated, multidisciplinary management targeting both gut inflammation and metabolic dysfunction is essential to improve long-term outcomes in this growing patient group.

Limitations

The evidence synthesis is constrained by a limited number of studies, predominantly cross-sectional in design, precluding causal inference. The temporal relationship between MASLD diagnosis and surgery, for example, is not explicit in any of the studies, which limits inference on causality or sequence. A formal assessment of publication bias was not performed due to the limited number of included studies. Heterogeneity in MASLD diagnostic criteria, metabolic phenotyping, and inconsistent reporting of fibrosis staging and IBD activity indices restricted deeper comparative analyses. These limitations underscore the need for large, prospective longitudinal cohorts.

## Conclusions

In patients with inflammatory bowel disease (IBD), the presence of metabolic dysfunction-associated steatotic liver disease (MASLD) is strongly associated with an increased prevalence of cardiometabolic comorbidities, including a greater than 12-fold higher odds of diabetes mellitus and a 2.6-fold higher odds of hypertension. In contrast, no significant association was observed between MASLD and the need for IBD-related surgery, suggesting that MASLD does not modify the severity or progression of intestinal disease. These observations indicate that MASLD in the context of IBD primarily reflects coexisting metabolic dysregulation rather than being solely a consequence of chronic gut inflammation. Consequently, systematic screening for cardiometabolic risk factors and early management of metabolic comorbidities are warranted in IBD patients diagnosed with MASLD. Larger prospective studies are needed to further clarify the nature and directionality of these associations and their long-term clinical implications.
